# Association Between Hormone-Modulating Breast Cancer Therapies and Incidence of Neurodegenerative Outcomes for Women With Breast Cancer

**DOI:** 10.1001/jamanetworkopen.2020.1541

**Published:** 2020-03-24

**Authors:** Gregory L. Branigan, Maira Soto, Leigh Neumayer, Kathleen Rodgers, Roberta Diaz Brinton

**Affiliations:** 1 Center for Innovation in Brain Science, University of Arizona, Tucson; 2 Department of Pharmacology, University of Arizona College of Medicine, Tucson; 3 MD-PhD Training Program, University of Arizona College of Medicine, Tucson; 4 Department of Surgery, University of Arizona College of Medicine, Tucson; 5 Department of Obstetrics and Gynecology, University of Arizona College of Medicine, Tucson; 6 Department of Neurology, University of Arizona College of Medicine, Tucson

## Abstract

**Importance:**

The association between exposure to hormone-modulating therapy (HMT) as breast cancer treatment and neurodegenerative disease (NDD) is unclear.

**Objective:**

To determine whether HMT exposure is associated with the risk of NDD in women with breast cancer.

**Design, Setting, and Participants:**

This retrospective cohort study used the Humana claims data set from January 1, 2007, to March 31, 2017. The Humana data set contains claims from private-payer and Medicare insurance data sets from across the United States with a population primarily residing in the Southeast. Patient claims records were surveyed for a diagnosis of NDD starting 1 year after breast cancer diagnosis for the duration of enrollment in the claims database. Participants were 57 843 women aged 45 years or older with a diagnosis of breast cancer. Patients were required to be actively enrolled in Humana claims records for 6 months prior to and at least 3 years after the diagnosis of breast cancer. The analyses were conducted between January 1 and 15, 2020.

**Exposure:**

Hormone-modulating therapy (selective estrogen receptor modulators, estrogen receptor antagonists, and aromatase inhibitors).

**Main Outcomes and Measures:**

Patients receiving HMT for breast cancer treatment were identified. Survival analysis was used to determine the association between HMT exposure and diagnosis of NDD. A propensity score approach was used to minimize measured and unmeasured selection bias.

**Results:**

Of the 326 485 women with breast cancer in the Humana data set between 2007 and 2017, 57 843 met the study criteria. Of these, 18 126 (31.3%; mean [SD] age, 76.2 [7.0] years) received HMT, whereas 39 717 (68.7%; mean [SD] age, 76.8 [7.0] years) did not receive HMT. Mean (SD) follow-up was 5.5 (1.8) years. In the propensity score–matched population, exposure to HMT was associated with a decrease in the number of women who received a diagnosis of NDD (2229 of 17 878 [12.5%] vs 2559 of 17 878 [14.3%]; relative risk, 0.89; 95% CI, 0.84-0.93; *P* < .001), Alzheimer disease (877 of 17 878 [4.9%] vs 1068 of 17 878 [6.0%]; relative risk, 0.82; 95% CI, 0.75-0.90; *P* < .001), and dementia (1862 of 17 878 [10.4%] vs 2116 of 17 878 [11.8%]; relative risk, 0.88; 95% CI, 0.83-0.93; *P* < .001). The number needed to treat was 62.51 for all NDDs, 93.61 for Alzheimer disease, and 69.56 for dementia.

**Conclusions and Relevance:**

Among patients with breast cancer, tamoxifen and steroidal aromatase inhibitors were associated with a decrease in the number who received a diagnosis of NDD, specifically Alzheimer disease and dementia.

## Key Points

**Question:**

Is hormone-modulating therapy associated with neurodegenerative disease in women with breast cancer?

**Findings:**

In this cohort study of 57 843 perimenopausal- to postmenopausal-aged women with breast cancer, exposure to hormone-modulating therapy (tamoxifen and aromatase inhibitors, especially exemestane) was associated with a significant decrease in the number of women who received a diagnosis of neurodegenerative disease, most specifically Alzheimer disease.

**Meaning:**

With the increased life expectancy seen after treatment, therapy selection for breast cancer should include a careful discussion of the risks and benefits of each treatment option that may be associated with a reduced risk of neurodegenerative disease.

## Teaser

This cohort study uses the Humana claims data set to examine whether exposure to hormone-modulating therapy is associated with the risk of neurodegenerative disease in women with breast cancer.

## Introduction

Worldwide, breast cancer is the second most common cancer in women (after skin cancer).^1^ Approximately 12.8% of women will receive a diagnosis of breast cancer during their lifetime.^1^ As of 2019, more than 268 000 new cases of breast cancer were diagnosed, representing 15.2% of all new cases of cancer.^2^ As of 2016, 3 477 866 women were estimated to be living with breast cancer in the United States,^3^ and the number of breast cancer cases continues to increase.^2^ Although the rate of death from breast cancer has decreased, the mean 5-year relative survival rate is 89.9% and ranges from 98.8% to 27.4%.^2^ As the number of women with a diagnosis of breast cancer increases and survival rates improve, the number of women living with breast cancer who are at risk for other diseases will escalate. Thus, the potential additional risks and benefits of the therapies to reduce breast cancer recurrence will have increasing importance.

One potential factor associated with the increase in new cases of breast cancer is an aging population. Age remains a major risk factor for breast cancer, with 61 years as the median age at diagnosis.^1^ In parallel, age is the greatest risk factor for developing age-associated neurodegenerative diseases (NDDs).^4,5^ Two age-associated NDDs have a greater prevalence among women: Alzheimer disease (AD) and multiple sclerosis (MS).^6,7^ Women are at a 2-fold greater lifetime risk than men for developing AD, and MS is 2.8 times more prevalent among women than men.^7,8^

Currently, AD affects 1 in 9 persons in the US older than 65 years,^9^ two-thirds of whom are women. In this age group, breast cancer is projected to increase and will account for almost one-third of all cancers among women in 2019.^1,2^ The improvement in survival after breast cancer is associated with the long-term use of antiestrogen therapies. Often these therapies are associated with subjective reports of diminished cognitive function, an early indicator of AD risk.^10,11^

Therapies for breast cancer include surgery, radiotherapy, hormonal modulation, biologics, and chemotherapy.^12^ Most breast cancers express estrogen and progesterone receptors (hormone positive) and generally respond well to surgery with or without radiotherapy and to systemic therapy with hormone modulation.^12^ Given the prevalence of hormone-positive breast cancer, there are multiple breast cancer therapies targeting the estrogen receptor or the production of estrogen.^12,13^ Hormone-modulating therapies (HMTs) include the selective estrogen receptor modulators (SERMs; tamoxifen and raloxifene) and aromatase inhibitors (steroidal, exemestane; nonsteroidal, anastrozole and letrozole). These drugs have been used for the treatment of estrogen receptor–positive breast cancers and have been shown to decrease estrogen’s effects at the level of the breast tissue.^13^ Tamoxifen is used in both the treatment and the prevention of estrogen receptor–positive breast cancer and is a common therapy for premenopausal women and an option for postmenopausal women.^12^

Analyses reported herein were designed to determine potential associations between HMT cancer therapies that affect estrogen’s action and the incidence of 4 age-associated NDDs: AD, MS, Parkinson disease, and amyotrophic lateral sclerosis. Our study was conducted using a US-based population electronic medical record data set and a substantially larger number of women with breast cancer than previously reported.^14,15,16,17,18,19^ Furthermore, we investigated the risk of developing multiple age-associated NDDs that occur sporadically within an aging population. We report the association of individual hormonal modulators and their drug families within the HMT category with the risk of development of age-associated NDDs.

## Methods

### Data Source

The Humana data set is an insurance claims data set that serves the United States, with a population primarily residing in the Southeastern region. PearlDiver is for-fee research software that facilitates interaction with individual commercial, state-based Medicaid, Medicare stand-alone prescription drug plan, group Medicare Advantage, and individual Medicare Advantage data sets.^20^ The Humana data set contains patient demographic characteristics, prescription records, and numerous other data points for patients with *Current Procedural Terminology*, *International Classification of Diseases, Ninth Revision*, and *International Statistical Classification of Diseases and Related Health Problems, Tenth Revision* codes. As of June 2018, Humana represented 25 million patients with claims, including prescription records, from January 1, 2007, through March 31, 2017. This report follows the Strengthening the Reporting of Observational Studies in Epidemiology (STROBE) reporting guideline. This study was approved by the University of Arizona Institutional Review Board. Requirements for informed consent were waived because the data were deidentified.

### Study Variables

The outcome variable was defined as the occurrence of the first NDD diagnosis for each outcome of interest based on *International Classification of Diseases, Ninth Revision, Clinical Modification* and *International Statistical Classification of Diseases and Related Health Problems, Tenth Revision, Procedure Coding System* codes in the patient’s medical claims data. The HMT exposure group is defined as patients having at least 1 medication charge occurring after the diagnosis of breast cancer. Age is defined by the age at diagnosis of breast cancer. Neurodegenerative diseases included AD, dementia, Parkinson disease, MS, and amyotrophic lateral sclerosis (eTable 4 in the Supplement). Special attention was given to comorbidities known to be associated with NDD outcomes: stroke, hypertension, cardiovascular disease, type 2 diabetes, and chronic kidney disease (eTable 4 in the Supplement). For the chemotherapy analysis, intravenous therapeutics were excluded (eTable 3 in the Supplement).

### Statistical Analysis

Statistical analyses were conducted between January 1 and 15, 2020. Patient demographic statistics and incidence statistics were analyzed using unpaired 2-tailed *t* tests or χ^2^ tests, as appropriate, to test the significance of the differences between continuous and categorical variables. In all analyses, a 2-sided *P* < .05 was considered statistically significant.

After the unadjusted analysis, a propensity score–matched population was generated using the Bellwether-PearlDiver Interface and analyzed again using the Fisher exact test. Specifically, the association of HMT with NDD and each subtype was estimated in the unadjusted populations. To minimize confounding by indication, we used propensity score analysis to examine the association between HMT and subsequent NDD (or subtype). For the propensity score matching, using logistic regression, we first estimated for each participant the probability (ie, the propensity) of receiving HMT based on age, race/ethnicity, comorbidities of interest (Table 1), and Charlson Comorbidity Index score. Next, we modeled the associations between NDD and HMT, weighted by the inverse propensity score, after adjusting for stroke and chronic obstructive pulmonary disease (COPD) as statistically significant values obtained from the linear regression analysis. To examine the effect of weighting, we compared the covariates before and after adjustment for propensity score.

**Table 1.** Baseline Characteristics for Unadjusted Enrolled and Propensity Score–Matched Patients With or Without HMT Exposure

Characteristic	Unadjusted cohort			Propensity score–matched cohort^a^		
	Patients, No. (%)		*P* Value	Patients, No. (%)		*P* value
	HMT (n = 18 126)	No HMT (n = 39 717)		HMT (n = 17 878)	No HMT (n = 17 878)	
Age, y						
45-49	660 (3.6)	1701 (4.3)	<.001	647 (3.6)	820 (4.6)	<.001
50-54	709 (3.9)	1871 (4.7)		686 (3.8)	833 (4.7)	
55-59	913 (5.0)	2184 (5.5)		893 (5.0)	966 (5.4)	
60-64	1123 (6.2)	2504 (6.3)		1103 (6.2)	1140 (6.4)	
65-69	4618 (25.5)	10040 (25.3)		4557 (25.5)	4552 (25.5)	
70-74	4426 (24.4)	8493 (21.4)		4373 (24.5)	3852 (21.6)	
75-79	2930 (16.2)	6083 (15.3)		2899 (16.2)	2709 (15.2)	
80-84	1699 (9.4)	3758 (9.5)		1686 (9.4)	1647 (9.2)	
85-89	320 (1.8)	767 (1.9)		314 (1.8)	333 (1.9)	
≥90	728 (4.0)	2316 (5.8)		720 (4.0)	1026 (5.7)	
Race/ethnicity						
Unknown	2051 (11.3)	4762 (12.0)	.04	1995 (11.2)	2208 (12.4)	.01
White	13 642 (75.3)	29 261 (73.7)		13 443 (75.2)	13 105 (73.3)	
Black	2000 (11.0)	4574 (11.5)		1969 (11.0)	2085 (11.7)	
Other	151 (0.8)	318 (0.8)		149 (0.8)	140 (0.8)	
Asian	95 (0.5)	213 (0.5)		93 (0.5)	99 (0.6)	
Hispanic	200 (1.1)	481 (1.2)		198 (1.1)	203 (1.1)	
North American Native	32 (0.2)	63 (0.2)		31 (0.2)	38 (0.2)	
Comorbidities						
Type 2 diabetes	1079 (6.0)	2188 (5.5)	.03	998 (5.6)	1030 (5.8)	.48
CVD	374 (2.1)	995 (2.5)	.01	370 (2.1)	423 (2.4)	.06
Hypertension	2459 (13.6)	5500 (13.9)	.37	2423 (13.6)	2438 (13.6)	.83
CKD	391 (2.2)	987 (2.5)	.02	388 (2.2)	417 (2.3)	.32
Stroke	355 (2.0)	904 (2.3)	.02	349 (2.0)	399 (2.2)	.07
COPD	184 (1.0)	569 (1.4)	<.001	183 (1.0)	235 (1.3)	.01
Charlson Comorbidity Index						
0-4	14 174 (78.2)	30 654 (77.2)	<.001	13 982 (78.2)	14 536 (81.3)	<.001
5-10	3647 (20.1)	8077 (20.3)		3599 (20.1)	3068 (17.2)	
≥11	280 (1.5)	869 (2.2)		297 (1.7)	274 (1.5)	

Abbreviations: CKD, chronic kidney disease; COPD, chronic obstructive pulmonary disease; CVD, cardiovascular disease; HMT, hormone-modulating therapy.

^a^ Adjusted for history of stroke and COPD before the diagnosis of breast cancer.

Kaplan-Meier curves were created using the propensity score–matched population generated using the Bellwether-PearlDiver Interface. Medication possession ratios were used to calculate the median adherence rates for each HMT type.

## Results

**Figure 1.**
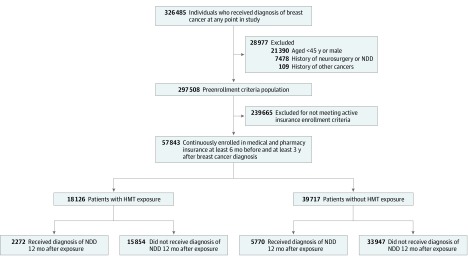
Study Design and Patient Breakdown HMT indicates hormone-modulating therapy; NDD, neurodegenerative disease.

Of the 326 485 patients with breast cancer in the Humana database, 57 843 met the inclusion and exclusion criteria and the claims enrollment period requirements for our study (Figure 1). An index date 1 year after the diagnosis of breast cancer was selected to rule out any diagnosis likely associated with chemotherapy or other interventions administered immediately after diagnosis before the start of HMT. Patient groups were defined according to the therapeutic intervention used. Of the 57 843 patients enrolled in the study, 18 126 (mean [SD] age, 76.2 [7.0] years) received HMT, whereas 39 717 individuals (mean [SD] age, 76.8 [7.0] years) were not treated with HMT (Figure 1). Hormone-modulating therapy was started a mean (SD) 133 (134) days after the diagnosis of breast cancer. The mean number of filled prescription days was 1078 (interquartile range, 540-1560). The drugs defined as HMT, the number of patients, and the median adherence rate for each drug are reported in eTable 1 in the Supplement. The generic drug codes used within the PearlDiver database are included in eTable 2 in the Supplement. These patient groups were then followed up for the duration of their claims data entries and surveyed for any diagnosis of NDD. The mean (SD) follow-up was 5.5 (1.8) years. The median (SD) time to diagnosis of NDD was 2.8 (2.3) years in the non-HMT exposure cohort and 2.9 (2.3) years in the HMT exposure cohort. The median (SD) time to diagnosis of AD was 3.1 (2.4) years in the non-HMT exposure cohort and 3.3 (2.2) years in the HMT exposure cohort.

The ages of patients included in the analysis ranged between 45 and 90 years of age or older, which was associated with significant differences in the age of patients receiving HMT vs no HMT. Most patient data records in the study were from women aged 55 to 69 years and women aged 80 to 89 years (Table 1). Furthermore, there were significant differences between the white patients who received HMT and white patients who did not receive HMT (13 642 of 18 126 [75.3%] vs 29 261 of 39 717 [73.7%]). Comorbidities were significantly different between patients who received HMT and those who did not (diabetes, 1079 of 18 126 [6.0%] vs 2188 of 39 717 [5.5%]; cardiovascular disease, 374 of 18 126 [2.1%] vs 995 of 39 717 [2.5%]; chronic kidney disease, 391 of 18 126 [2.2%] vs 987 of 39 717 [2.5%]; stroke, 355 of 18 126 [2.0%] vs 904 of 39 717 [2.3%]; COPD, 184 of 18 126 [1.0%] vs 569 of 39 717 [1.4%]). Last, there were significant differences between patients who received HMT and those who did not in Charlson Comorbidity Index score categories of 0 to 4 (14 174 of 18 126 [78.2%] vs 30 654 of 39 717 [77.2%]) and 11 or more (280 of 18 126 [1.5%] vs 869 of 39 717 [2.2%]). The linear regression of the comorbidities of interest and therapy selection indicated significant differences in therapy selection for those with a diagnosis of COPD and stroke. To address the association of comorbidities, propensity score matching was performed to create representative groups that controlled for COPD and stroke history. The demographic characteristics of the populations generated by propensity matching appear in Table 1 and show the same statistical differences between treatment groups as in the unadjusted data.

Analyses of unadjusted population data indicated that HMT exposure compared with no HMT exposure was associated with a significant decrease in the incidence of AD (900 of 18 126 [5.0%] vs 2436 of 39 717 [6.1%]; relative risk [RR], 0.81; 95% CI, 0.75-0.87; *P* < .001), dementia (1894 of 18 126 [10.4%] vs 4892 of 39 717 [12.3%]; RR, 0.85; 95% CI, 0.80-0.89; *P* < .001), non-AD dementia (1079 of 18 126 [6.0%] vs 2657 of 39 717 [6.7%]; RR, 0.89; 95% CI, 0.83-0.89; *P* < .001), and all NDDs (2272 of 18 126 [12.5%] vs 5770 of 39 717 [14.5%]; RR, 0.86; 95% CI, 0.82-0.90; *P* < .001) (Table 2). The outcomes of multiple sclerosis and Parkinson disease were not significantly different among those with HMT exposure. Although not significant, the incidence of amyotrophic lateral sclerosis appeared to be increased among patients exposed to HMT. No change was observed in the association of risk reduction with HMT in the unadjusted population after the removal of patients who received intravenous chemotherapy, which indicates that the association is likely due to the presence of the HMT and not to the cytotoxic effects of chemotherapy (eTable 3, eTable 5, and eFigure 2 in the Supplement).

**Table 2.** Relative Risk of Unadjusted and Propensity Score–Matched Patients Developing NDDs After Receiving HMT

Characteristic	All NDDs	AD	Dementia	Non-AD Dementia	MS	PD	ALS
Unadjusted cohort							
Patients who received HMT,^a^ No. (%)	2272 (12.5)	900 (5.0)	1894 (10.5)	1079 (6.0)	129 (0.7)	328 (1.8)	15 (0.1)
Patients who did not receive HMT,^b^ No. (%)	5770 (14.5)	2436 (6.1)	4892 (12.3)	2657 (6.7)	306 (0.8)	755 (1.9)	22 (0.1)
Relative risk (95% CI)	0.86 (0.82-0.90)	0.81 (0.75-0.87)	0.85 (0.80-0.89)	0.89 (0.83-0.89)	0.92 (0.75-1.13)	0.95 (0.84-1.08)	1.49 (0.78-2.85)
NNT	50.17	85.61	53.53	137.21	1702	1094	3655
* P* value	<.001	<.001	<.001	<.001	.46	.47	.29
Propensity score–matched cohort^c^							
Patients who received HMT,^a^ No. (%)	2229 (12.5)	877 (4.9)	1862 (10.4)	1040 (5.8)	NA	NA	NA
Patients who did not receive HMT,^b^ No. (%)	2559 (14.3)	1068 (6.0)	2116 (11.8)	1106 (6.2)	NA	NA	NA
Relative risk (95% CI)	0.89 (0.84-0.93)	0.82 (0.75-0.90)	0.88 (0.83-0.93)	0.94 (0.87-1.02)	NA	NA	NA
NNT	62.51	93.61	69.56	255.4	NA	NA	NA
* P* value	<.001	<.001	<.001	.15	NA	NA	NA

Abbreviations: AD, Alzheimer disease; ALS, amyotrophic lateral sclerosis; HMT, hormone-modulating therapy; MS, multiple sclerosis; NA, not applicable; NDD, neurodegenerative disease; NNT, number needed to treat; PD, Parkinson disease.

^a^ Unadjusted cohort, 18 126 patients; propensity score–matched cohort, 17 878 patients.

^b^ Unadjusted cohort, 39 717 patients; propensity score–matched cohort, 17 878 patients.

^c^ Adjusted for history of stroke and chronic obstructive pulmonary disease before the diagnosis of breast cancer.

In the propensity score–matched population, AD- and dementia-associated outcomes were specifically analyzed because these diagnoses were statistically significant in the unadjusted populations (Table 2). The results of the χ^2^ analysis in the matched patient group indicated that the significant decreases in the numbers of patients with a diagnosis of AD (877 of 17 878 [4.9%] vs 1068 of 17 878 [6.0%]; RR, 0.82; 95% CI, 0.75-0.90), dementia (1862 of 17 878 [10.4%] vs 2116 of 17 878 [11.8%]; RR, 0.88; 95% CI, 0.83-0.93; *P* < .001), and all NDDs ((2229 of 17 878 [12.5%] vs 2559 of 17 878 [14.3%]; RR, 0.89; 95% CI, 0.84-0.93; *P* < .001) who received HMT were sustained. The analysis of non-AD dementia outcomes, such as vascular dementia or Lewy body dementia, was no longer significant in the matched population. The propensity score–matched population was then used to generate Kaplan-Meier survival curves for NDD-free survival for each NDD subtype to evaluate the rate and percentage of the population who developed each disease (eFigure 1 in the Supplement). Changes in the rate of disease incidence between patients receiving HMT and patients not receiving HMT mirror the results seen in the χ^2^ analysis.

**Figure 2.**
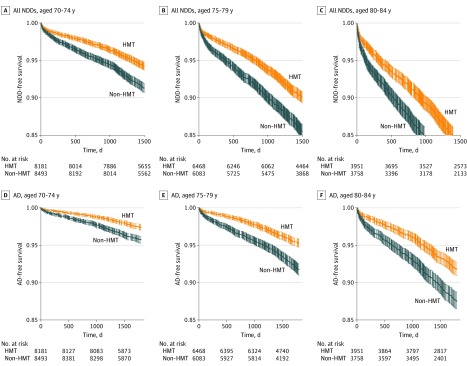
Age-Dependent Reduction in Risk for All Neurodegenerative Diseases (NDDs) and Alzheimer Disease (AD) Associated With Hormone-Modulating Therapy (HMT) Exposure A significantly decreased risk of diagnosis of both overall NDDs and, more specifically, AD was observed for patients treated with HMT vs those not treated with HMT.

In the propensity score–matched population, groups were stratified by age (65-69, 70-74, 75-79, and 80-84 years) to determine a potential age-specific association in overall NDD and AD risk outcomes (Figure 2; eFigure 3 in the Supplement). For patients 65 to 69 years of age, there was no significant difference between patients receiving HMT and patients not receiving HMT in the risk of NDD or AD; SDs overlapped in the 5-year analysis. In contrast, increasing age was associated with a greater reduction of risk for all NDDs in women receiving HMT; SDs did not overlap in the 5-year analysis and were divergent. This association between age and reduced incidence of NDD was replicated in the survival curves specifically for AD (Figure 2; eFigure 3 in the Supplement).

**Figure 3.**
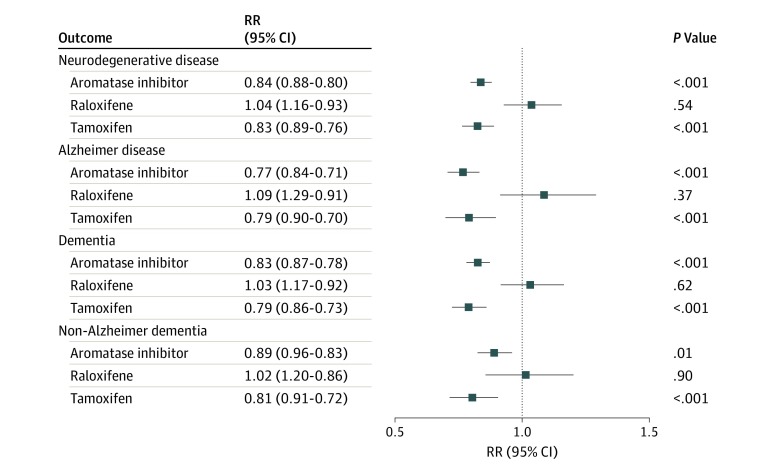
Relative Risk (RR) of All Neurodegenerative Disease, Alzheimer Disease, and Dementia Outcomes With Aromatase Inhibitors, Raloxifene, and Tamoxifen Tamoxifen and the aromatase inhibitors were associated with significantly reduced RRs for all neurodegenerative diseases, Alzheimer disease, dementia, and non-Alzheimer dementia in the hormone-modulating therapy treatment groups. More specifically, the steroidal aromatase inhibitor (exemestane) had a greater association than nonsteroidal aromatase inhibitors (anastrozole and letrozole) with reduced RR of neurodegenerative disease outcomes.

To address the potential selectivity of the action of HMT to reduce the incidence of AD, analysis of the incidence of AD by therapeutic mechanism of action and tissue specificity was conducted. Patients receiving HMT were divided into 3 groups based on therapeutic mechanism (Figure 3): tamoxifen (n = 5335), raloxifene (n = 1972), or aromatase inhibitors (n = 16 032). Tamoxifen showed the strongest associated decreased risk for each disease (RR, 0.84; 95% CI, 0.80-0.88; *P* < .001). Raloxifene, while also a SERM, had no significant association with the RR for any NDD (RR, 1.04; 95% CI, 0.93-1.16; *P* = .54). The aromatase inhibitors, known to block the enzyme responsible for the conversion of testosterone and androstenedione to estrogen,^21^ also was associated with a reduction in the RR for the development of the NDDs of interest (RR, 0.83; 95% CI, 0.76-0.89; *P* < .001). Thus, the reduced RR seen in the HMT-treated population is primarily associated with patients receiving tamoxifen or aromatase inhibitors (Figure 3).

Aromatase inhibitors are either nonsteroidal (anastrozole and letrozole) or steroidal (exemestane), which led to questions surrounding possible differences in their potential protective effects seen in AD and dementia (eFigure 4 in the Supplement). To address this question, analysis of the individual drugs in these classes was undertaken to determine potential differences in their association with the reduction of NDD risk. Outcomes of this analysis indicated that patients receiving exemestane, the steroidal aromatase inhibitor, had a statistically significant decrease in the incidence of AD and dementia compared with patients receiving the nonsteroidal drugs anastrozole and letrozole (eFigure 4 in the Supplement). However, both types of aromatase inhibitors exerted a protective association compared with patients with breast cancer who were not receiving any HMT.

## Discussion

The first goal of the National Plan to Address Alzheimer Disease is to prevent AD by 2025.^22^ The short time horizon for achieving this goal is challenging but not insurmountable. One potentially effective strategy is to identify populations of individuals at risk for AD who have received therapeutic interventions that modify the risk of a diagnosis of AD. Although complex, multiple epidemiologic studies indicate that estrogen hormone therapy is associated with a reduced risk of AD.^19,23,24,25,26,27,28,29,30,31,32^ The loss of estrogen in the brain can be a factor associated with the 2-fold greater lifetime risk of developing AD.^33^ The patient group in our study is one such group, women receiving antiestrogen therapies in midlife and later life.

Treatments for age-associated NDDs remain an unmet need and challenge because each NDD has a complex and multifaceted pathophysiology, resulting in few therapeutic options for treatment or prevention. For AD, HMTs (such as estrogen therapy) have been shown to be associated with the onset of AD,^34^ whereas estrogen therapy has been shown to be ineffective as a treatment for those with a diagnosis of AD.^35,36,37,38,39,40,41,42,43^ Similarly, the failure of trials evaluating the use of SERMs in the treatment of AD could be due to the fact that the intervention was started past the therapeutic window for HMTs.^18,44,45,46^ Here, we show the beneficial effects of exposure to HMT as a prophylactic treatment for the potential prevention of AD.

Although our preliminary results show a decrease in the number of NDDs overall in patients receiving HMTs, the predominant association was a significant decrease in the RR of dementia-related outcomes. The lack of significance in nondementia NDDs is likely owing to a decreased incidence of MS, Parkinson disease, and amyotrophic lateral sclerosis in our study population as well as in the general population overall. In the dementia-associated diseases, there was a larger association observed for AD outcomes despite the disease being less prevalent than non-AD dementias. This trend was also evident in the propensity score–matched populations, in which, after controlling for cerebrovascular and respiratory disease, the protective effects of HMT occurred exclusively for AD outcomes. This finding points to a potential specific biological mechanism associated with estrogen loss in the brain in the pathophysiology of AD. Alternatively, the results from the propensity score–matched populations could be due to a stronger association of cerebrovascular and respiratory disease with non-AD dementia.

Previous reports have typically focused on a single drug and disease outcome, which limits the scope of the translational outcomes. We have included all HMTs used in breast cancer treatment as well as multiple age-associated NDDs. These treatments generally fall into 2 classes: SERMs and aromatase inhibitors. Tamoxifen and aromatase inhibitors exhibited the strongest association with reducing the incidence of AD and related dementia. The protection associated with the SERMs was exclusively due to tamoxifen and not to raloxifene. This finding might explain why previous studies using SERMs were not found to be effective because raloxifene was the focus of several of these AD trials.^18,47^ Mechanistically, tamoxifen and raloxifene are known to act in a tissue-specific manner. Tamoxifen and raloxifene are known estrogen receptor antagonists in breast tissue but show divergent actions in uterine tissue^48,49,50,51^ and brain tissue.^52,53^ Aromatase inhibitors are known to act systemically to decrease the amount of estrogen.^21^ However, a recent study suggests that there also may be divergent actions of aromatase inhibitors in specific brain regions.^54^ Alternatively, upstream precursors of estrogen, such as testosterone and androstenedione, which may be increased by aromatase inhibitors, can be associated with cognition.^55,56^ If tamoxifen and aromatase inhibitors are acting to increase estrogen-related actions in brain tissue, the argument for the protective association of estrogen with AD-related outcomes is strengthened.

### Limitations

This analysis has several limitations. First, it is a retrospective analysis of a claims database. The patients included may have obtained services outside of those included in this database. Second, there could be factors, known and unknown, that even with propensity matching may not be adequately addressed. Third, the rate of women with a diagnosis of breast cancer who were exposed to HMT is seemingly low (approximately one-third of the sample). Although this proportion seems low, there are other data that show that, while adherence to endocrine therapy in clinical trials is high, adherence in clinical practice is substantially lower, with only about 50% of women completing 5 years of therapy.^57^ The factors associated with nonadherence include perception of a low risk of recurrence, adverse effects (perceived or real), costs, suboptimal patient-physician communication, and lack of social support.^57^ Moreover, in the age-stratified data (Figure 2; eFigure 3 in the Supplement), we show that a low level of HMT exposure was associated with the population younger than 70 years (8237 of 26 627 patients aged <70 years received HMT [31.2%]), whereas patients 70 years or older received HMT at a rate of 50% (18 600 of 36 934). In addition, HMT exposure is assessed by filled prescription charges to Humana, indicating that a drug has been picked up by a patient; however, data on specific breast pathologic condition, on contraindications for therapy, and on therapeutics actually prescribed for a patient cannot be assessed in this data set.

## Conclusions

This study found that among patients with breast cancer, tamoxifen and steroidal aromatase inhibitors were associated with a decrease in the number who received a diagnosis of NDD, specifically AD and dementia. As we advance in our abilities to prevent, treat, and cure cancer, discussions around optimal care will need to include understanding the long-term outcomes of therapy selection for age-related NDDs. The fact that breast cancer is the second most common cancer in women (after skin cancer) and that women are disproportionately affected by AD and related dementia provides us with an opportunity to reduce the global disease burden of NDDs.^58^

## Notes

Supplement. **eTable 1.** List of HMT and Number of Patients Taking HMT **eTable 2.** List of Hormone Modulating Therapy Drug Codes Used **eTable 3.** List of Chemotherapy Drug Codes Used **eTable 4.** List of Diagnose Codes Used **eFigure 1.** Reduced Risk of All NDD, AD, and Dementia in Propensity Score–Matched Patients With HMT Exposure Across All Age Groups **eFigure 2.** Patient Breakdown Without Chemotherapy **eTable 5.** Relative Risk of Patients Without Chemotherapy Taking HMT to Develop NDDs **eFigure 3.** Age-Dependent Reduction in Risk for All NDD and Alzheimer Disease Is Not Associated With HMT Exposure for Patients Less Than 70 Years of Age **eFigure 4.** Steroidal Aromatase Inhibitors Are Associated With the Protective Effects Seen Within the Aromatase Inhibitor Group

## References

[1] Siegel RL, Miller KD, Jemal A Cancer statistics, 2019. CA Cancer J Clin. 2019;69(1):-. doi:10.3322/caac.21551 30620402

[2] Surveillance, Epidemiology, and End Results Program. SEER cancer statistics review (CSR) 1975-2016. Accessed January 1, 2020. https://seer.cancer.gov/csr/1975_2016/

[3] Rojas K, Stuckey A Breast cancer epidemiology and risk factors. Clin Obstet Gynecol. 2016;59(4):651-672. doi:10.1097/GRF.0000000000000239 27681694

[4] Johnson IP Age-related neurodegenerative disease research needs aging models. Front Aging Neurosci. 2015;7:168. doi:10.3389/fnagi.2015.00168 26388766 PMC4557095

[5] Niccoli T, Partridge L Ageing as a risk factor for disease. Curr Biol. 2012;22(17):R741-R752. doi:10.1016/j.cub.2012.07.024 22975005

[6] Riedel BC, Thompson PM, Brinton RD Age, *APOE* and sex: triad of risk of Alzheimer’s disease. J Steroid Biochem Mol Biol. 2016;160:134-147. doi:10.1016/j.jsbmb.2016.03.012 26969397 PMC4905558

[7] Wallin MT, Culpepper WJ, Campbell JD, ; US Multiple Sclerosis Prevalence Workgroup The prevalence of MS in the United States: a population-based estimate using health claims data. Neurology. 2019;92(10):e1029-e1040. doi:10.1212/WNL.0000000000007035 30770430 PMC6442006

[8] Noonan CW, Kathman SJ, White MC Prevalence estimates for MS in the United States and evidence of an increasing trend for women. Neurology. 2002;58(1):136-138. doi:10.1212/WNL.58.1.136 11781421

[9] Alzheimer's Association 2018 Alzheimer’s disease facts and figures. Alzheimers Dement. 2018;14(3):367-429. doi:10.1016/j.jalz.2018.02.001

[10] Biro E, Kahan Z, Kalman J, Cognitive functioning and psychological well-being in breast cancer patients on endocrine therapy. In Vivo. 2019;33(4):1381-1392. doi:10.21873/invivo.11615 31280234 PMC6689374

[11] Nead KT, Gaskin G, Chester C, Swisher-McClure S, Leeper NJ, Shah NH Association between androgen deprivation therapy and risk of dementia. JAMA Oncol. 2017;3(1):49-55. doi:10.1001/jamaoncol.2016.3662 27737437

[12] National Comprehensive Cancer Network Practice guidelines in oncology: breast cancer: version 2.2017. Accessed September 15, 2019. https://www.nccn.org/professionals/physician_gls/pdf/breast_blocks.pdf

[13] Chen WY Selective estrogen receptor modulators and aromatase inhibitors for breast cancer prevention. UpToDate. Accessed July 31, 2019. https://www.uptodate.com/contents/selective-estrogen-receptor-modulators-and-aromatase-inhibitors-for-breast-cancer-prevention

[14] Sun LM, Chen HJ, Liang JA, Kao CH Long-term use of tamoxifen reduces the risk of dementia: a nationwide population-based cohort study. QJM. 2016;109(2):103-109. doi:10.1093/qjmed/hcv072 25852154

[15] Liao KF, Lin CL, Lai SW Nationwide case-control study examining the association between tamoxifen use and Alzheimer’s disease in aged women with breast cancer in Taiwan. Front Pharmacol. 2017;8:612. doi:10.3389/fphar.2017.00612 28928665 PMC5591818

[16] Latourelle JC, Dybdahl M, Destefano AL, Myers RH, Lash TL Risk of Parkinson’s disease after tamoxifen treatment. BMC Neurol. 2010;10:23. doi:10.1186/1471-2377-10-23 20385012 PMC2862029

[17] Kesler SR, Rao V, Ray WJ, Rao A; Alzheimer’s Disease Neuroimaging Initiative Probability of Alzheimer’s disease in breast cancer survivors based on gray-matter structural network efficiency. Alzheimers Dement (Amst). 2017;9:67-75. doi:10.1016/j.dadm.2017.10.002 29201992 PMC5700833

[18] Henderson VW, Ala T, Sainani KL, Raloxifene for women with Alzheimer disease: a randomized controlled pilot trial. Neurology. 2015;85(22):1937-1944. doi:10.1212/WNL.0000000000002171 26537053 PMC4664126

[19] Tang M-X, Jacobs D, Stern Y, Effect of oestrogen during menopause on risk and age at onset of Alzheimer’s disease. Lancet. 1996;348(9025):429-432. doi:10.1016/S0140-6736(96)03356-9 8709781

[20] PearlDiver. Healthcare research. Accessed February 11, 2020. http://www.pearldiverinc.com

[21] Miller WR Aromatase inhibitors: mechanism of action and role in the treatment of breast cancer. Semin Oncol. 2003;30(4)(suppl 14):3-11. doi:10.1016/S0093-7754(03)00302-6 14513432

[22] Office of the Assistant Secretary for Planning and Evaluation National plan to address Alzheimer’s disease: 2018 update. Accessed January 1, 2020. https://aspe.hhs.gov/report/national-plan-address-alzheimers-disease-2018-update

[23] Cholerton B, Gleason CE, Baker LD, Asthana S Estrogen and Alzheimer’s disease: the story so far. Drugs Aging. 2002;19(6):405-427. doi:10.2165/00002512-200219060-00002 12149049

[24] Fillit H, Cummings J; Alzheimer’s Disease (AD) Managed Care Advisory Council Practice guidelines for the diagnosis and treatment of Alzheimer’s disease in a managed care setting: part II—pharmacologic therapy. Manag Care Interface. 2000;13(1):51-56. 10747691

[25] Fillit HM The role of hormone replacement therapy in the prevention of Alzheimer disease. Arch Intern Med. 2002;162(17):1934-1942. doi:10.1001/archinte.162.17.1934 12230415

[26] Janicki SC, Schupf N Hormonal influences on cognition and risk for Alzheimer’s disease. Curr Neurol Neurosci Rep. 2010;10(5):359-366. doi:10.1007/s11910-010-0122-6 20535591 PMC3058507

[27] Merlo S, Spampinato SF, Sortino MA Estrogen and Alzheimer’s disease: still an attractive topic despite disappointment from early clinical results. Eur J Pharmacol. 2017;817:51-58. doi:10.1016/j.ejphar.2017.05.059 28577965

[28] Mulnard RA, Cotman CW, Kawas C, Estrogen replacement therapy for treatment of mild to moderate Alzheimer disease: a randomized controlled trial: Alzheimer’s Disease Cooperative Study. JAMA. 2000;283(8):1007-1015. doi:10.1001/jama.283.8.1007 10697060

[29] Simpkins JW, Perez E, Wang X, Yang S, Wen Y, Singh M The potential for estrogens in preventing Alzheimer’s disease and vascular dementia. Ther Adv Neurol Disord. 2009;2(1):31-49. doi:10.1177/1756285608100427 19890493 PMC2771945

[30] Villa A, Vegeto E, Poletti A, Maggi A Estrogens, neuroinflammation, and neurodegeneration. Endocr Rev. 2016;37(4):372-402. doi:10.1210/er.2016-1007 27196727 PMC4971309

[31] Zandi PP, Carlson MC, Plassman BL, ; Cache County Memory Study Investigators Hormone replacement therapy and incidence of Alzheimer disease in older women: the Cache County Study. JAMA. 2002;288(17):2123-2129. doi:10.1001/jama.288.17.2123 12413371

[32] Zhao L, O’Neill K, Brinton RD Estrogenic agonist activity of ICI 182,780 (Faslodex) in hippocampal neurons: implications for basic science understanding of estrogen signaling and development of estrogen modulators with a dual therapeutic profile. J Pharmacol Exp Ther. 2006;319(3):1124-1132. doi:10.1124/jpet.106.109504 16951259

[33] Brinton RD, Yao J, Yin F, Mack WJ, Cadenas E Perimenopause as a neurological transition state. Nat Rev Endocrinol. 2015;11(7):393-405. doi:10.1038/nrendo.2015.82 26007613 PMC9934205

[34] Fontes F, Pereira S, Castro-Lopes JM, Lunet N A prospective study on the neurological complications of breast cancer and its treatment: updated analysis three years after cancer diagnosis. Breast. 2016;29:31-38. doi:10.1016/j.breast.2016.06.013 27394676

[35] Sherwin BB Estrogen and cognitive functioning in women: lessons we have learned. Behav Neurosci. 2012;126(1):123-127. doi:10.1037/a0025539 22004260 PMC4838456

[36] Shumaker SA, Legault C, Rapp SR, ; WHIMS Investigators Estrogen plus progestin and the incidence of dementia and mild cognitive impairment in postmenopausal women: the Women’s Health Initiative Memory Study: a randomized controlled trial. JAMA. 2003;289(20):2651-2662. doi:10.1001/jama.289.20.2651 12771112

[37] McCarrey AC, Resnick SM Postmenopausal hormone therapy and cognition. Horm Behav. 2015;74:167-172. doi:10.1016/j.yhbeh.2015.04.018 25935728 PMC4573348

[38] Rapp SR, Espeland MA, Shumaker SA, ; WHIMS Investigators Effect of estrogen plus progestin on global cognitive function in postmenopausal women: the Women’s Health Initiative Memory Study: a randomized controlled trial. JAMA. 2003;289(20):2663-2672. doi:10.1001/jama.289.20.2663 12771113

[39] Chen S, Nilsen J, Brinton RD Dose and temporal pattern of estrogen exposure determines neuroprotective outcome in hippocampal neurons: therapeutic implications. Endocrinology. 2006;147(11):5303-5313. doi:10.1210/en.2006-0495 16916950

[40] Breuer B, Anderson R The relationship of tamoxifen with dementia, depression, and dependence in activities of daily living in elderly nursing home residents. Women Health. 2000;31(1):71-85. doi:10.1300/J013v31n01_05 11005221

[41] Vogelvang TE, Mijatovic V, van der Mooren MJ, Effect of raloxifene and hormone therapy on serum markers of brain and whole-body cholesterol metabolism in postmenopausal women. Maturitas. 2005;50(4):312-320. doi:10.1016/j.maturitas.2004.08.004 15780532

[42] Chen X, He X, Tao L, The working memory and dorsolateral prefrontal-hippocampal functional connectivity changes in long-term survival breast cancer patients treated with tamoxifen. Int J Neuropsychopharmacol. 2017;20(5):374-382. doi:10.1093/ijnp/pyx008 28177081 PMC5417059

[43] Le Rhun E, Delbeuck X, Lefeuvre-Plesse C, A phase III randomized multicenter trial evaluating cognition in post-menopausal breast cancer patients receiving adjuvant hormonotherapy. Breast Cancer Res Treat. 2015;152(3):569-580. doi:10.1007/s10549-015-3493-1 26160250

[44] Barron TI, Connolly R, Bennett K, Feely J, Kennedy MJ Early discontinuation of tamoxifen: a lesson for oncologists. Cancer. 2007;109(5):832-839. doi:10.1002/cncr.22485 17243168

[45] Hochner-Celnikier D Pharmacokinetics of raloxifene and its clinical application. Eur J Obstet Gynecol Reprod Biol. 1999;85(1):23-29. doi:10.1016/S0301-2115(98)00278-4 10428318

[46] Nickelsen T, Lufkin EG, Riggs BL, Cox DA, Crook TH Raloxifene hydrochloride, a selective estrogen receptor modulator: safety assessment of effects on cognitive function and mood in postmenopausal women. Psychoneuroendocrinology. 1999;24(1):115-128. doi:10.1016/S0306-4530(98)00041-9 10098223

[47] Yaffe K Estrogens, selective estrogen receptor modulators, and dementia: what is the evidence? Ann N Y Acad Sci. 2001;949:215-222. doi:10.1111/j.1749-6632.2001.tb04024.x 11795356

[48] Cano A, Hermenegildo C The endometrial effects of SERMs. Hum Reprod Update. 2000;6(3):244-254. doi:10.1093/humupd/6.3.244 10874569

[49] Hu R, Hilakivi-Clarke L, Clarke R Molecular mechanisms of tamoxifen-associated endometrial cancer. [review]. Oncol Lett. 2015;9(4):1495-1501. doi:10.3892/ol.2015.2962 25788989 PMC4356269

[50] Polin SA, Ascher SM The effect of tamoxifen on the genital tract. Cancer Imaging. 2008;8(1):135-145. doi:10.1102/1470-7330.2008.0020 18603495 PMC2482152

[51] Rey JR, Cervino EV, Rentero ML, Crespo EC, Alvaro AO, Casillas M Raloxifene: mechanism of action, effects on bone tissue, and applicability in clinical traumatology practice. Open Orthop J. 2009;3:14-21. doi:10.2174/1874325000903010014 19516920 PMC2687107

[52] O’Neill K, Chen S, Brinton RD Impact of the selective estrogen receptor modulator, raloxifene, on neuronal survival and outgrowth following toxic insults associated with aging and Alzheimer’s disease. Exp Neurol. 2004;185(1):63-80. doi:10.1016/j.expneurol.2003.09.005 14697319

[53] O’Neill K, Chen S, Diaz Brinton R Impact of the selective estrogen receptor modulator, tamoxifen, on neuronal outgrowth and survival following toxic insults associated with aging and Alzheimer’s disease. Exp Neurol. 2004;188(2):268-278. doi:10.1016/j.expneurol.2004.01.014 15246826

[54] Gervais NJ, Remage-Healey L, Starrett JR, Pollak DJ, Mong JA, Lacreuse A Adverse effects of aromatase inhibition on the brain and behavior in a nonhuman primate. J Neurosci. 2019;39(5):918-928. doi:10.1523/JNEUROSCI.0353-18.2018 30587540 PMC6382974

[55] Barrett-Connor E, Goodman-Gruen D Cognitive function and endogenous sex hormones in older women. J Am Geriatr Soc. 1999;47(11):1289-1293. doi:10.1111/j.1532-5415.1999.tb07427.x 10573435

[56] Barrett-Connor E, Goodman-Gruen D, Patay B Endogenous sex hormones and cognitive function in older men. J Clin Endocrinol Metab. 1999;84(10):3681-3685. doi:10.1210/jc.84.10.3681 10523014

[57] Chlebowski RT, Kim J, Haque R Adherence to endocrine therapy in breast cancer adjuvant and prevention settings. Cancer Prev Res (Phila). 2014;7(4):378-387. doi:10.1158/1940-6207.CAPR-13-0389 24441675 PMC11649036

[58] Cummings J, Lee G, Ritter A, Zhong K Alzheimer’s disease drug development pipeline: 2018. Alzheimers Dement (N Y). 2018;4:195-214. 29955663 10.1016/j.trci.2018.03.009 PMC6021548

