# Supplementary material for: Association Between Hormone-Modulating Breast Cancer Therapies and Incidence of Neurodegenerative Outcomes for Women With Breast Cancer
Source: JAMA Netw Open. 2020 Mar 24;3(3):e201541. doi: 10.1001/jamanetworkopen.2020.1541 (PMC7093781; doi:10.1001/jamanetworkopen.2020.1541)
Supplement: Supplement. — eTable 1. List of HMT and Number of Patients Taking HMT eTable 2. List of Hormone Modulating Therapy Drug Codes Used eTable 3. List of Chemotherapy Drug Codes Used eTable 4. List of Diagnose Codes Used eFigure 1. Reduced Risk of All NDD, AD, and Dementia in Propensity Score–Matched Patients With HMT Exposure Across All Age Groups eFigure 2. Patient Breakdown Without Chemotherapy eTable 5. Relative Risk of Patients Without Chemotherapy Taking HMT to Develop NDDs eFigure 3. Age-Dependent Reduction in Risk for All NDD and Alzheimer Disease Is Not Associated With HMT Exposure for Patients Less Than 70 Years of Age eFigure 4. Steroidal Aromatase Inhibitors Are Associated With the Protective Effects Seen Within the Aromatase Inhibitor Group [file jamanetwopen-3-e201541-s001.pdf]

# Supplementary Online Content

Branigan GL, Soto M, Neumayer L, Rodgers K, Brinton RD. Association between hormone-modulating breast cancer therapies and incidence of neurodegenerative outcomes for women with breast cancer. *JAMA Netw Open.* 2020;3(3):e201541. doi:10.1001/jamanetworkopen.2020.1541

**eTable 1.** List of HMT and Number of Patients Taking HMT

**eTable 2.** List of Hormone Modulating Therapy Drug Codes Used

**eTable 3.** List of Chemotherapy Drug Codes Used

**eTable 4.** List of Diagnose Codes Used

**eFigure 1.** Reduced Risk of All NDD, AD, and Dementia in Propensity Score–Matched Patients With HMT Exposure Across All Age Groups

**eFigure 2.** Patient Breakdown Without Chemotherapy

**eTable 5.** Relative Risk of Patients Without Chemotherapy Taking HMT to Develop NDDs

**eFigure 3.** Age-Dependent Reduction in Risk for All NDD and Alzheimer Disease Is Not Associated With HMT Exposure for Patients Less Than 70 Years of Age

**eFigure 4.** Steroidal Aromatase Inhibitors Are Associated With the Protective Effects Seen Within the Aromatase Inhibitor Group

This supplementary material has been provided by the authors to give readers additional information about their work.

**eTable 1.** List of HMT and Number of Patients Taking HMT

| Drug | n (%) | Median % Adherence | Average Daily Dose | Median Time Between BCA Diagnosis and Start of Therapy (days) |
|---|---|---|---|---|
| Raloxifene Hydrochloride | 1,972 (10.88) | 86.86% | 59.92 | 75 |
| Tamoxifen Citrate | 5,335 (29.43) | 89.50% | 19.96 | 188 |
| Tamoxifen, other | 0 | n/a | n/a | n/a |
| Anastrozole | 9,819 (54.17) | 94.68% | 1 | 125 |
| Letrozole | 4,663 (25.72) | 92.92% | 2.5 | 126 |
| Fulvestrant | 33 (0.18) | 91.38% | 18.07 | 1000 |
| Exemestane | 1,550 (8.55) | 93.46% | 25 | 465 |

© 2020 Branigan GL et al. *JAMA Network Open.*

**eTable 2.** List of Hormone Modulating Therapy Drug Codes Used

| **Drug** | |
|---|---|
| Raloxifene Hydrochloride | GENERIC_DRUG-100578 |
| Tamoxifen Citrate | GENERIC_DRUG-100858 |
| Tamoxifen, other | GENERIC_DRUG-103413 |
| Anastrozole | GENERIC_DRUG-101513 |
| Letrozole | GENERIC_DRUG-104120 |
| Fulvestrant | GENERIC_DRUG-105312 |
| Exemestane | GENERIC_DRUG-105517 |

© 2020 Branigan GL et al. *JAMA Network Open.*

**eTable 3.** List of Chemotherapy Drug Codes Used

| Drug | |
|---|---|
| Trastuzumab | GENERIC_DRUG-108717 |
| Epirubicin | GENERIC_DRUG-102011 |
| Fluorouracil | GENERIC_DRUG-101933, GENERIC_DRUG-104504, GENERIC_DRUG-104268 |
| Paclitaxel | GENERIC_DRUG-100259 |
| Vinblastine | GENERIC_DRUG-102905 |
| Doxorubicin | GENERIC_DRUG-102078 |
| Daunorubicin | GENERIC_DRUG-107513, GENERIC_DRUG-107300 |
| Gemcitabine | GENERIC_DRUG-102442, GENERIC_DRUG-101042 |
| Thiotepa | GENERIC_DRUG-110262 |
| Capecitabine | GENERIC_DRUG-103321 |
| Carboplatin | GENERIC_DRUG-104932 |
| Cyclophosphamide | GENERIC_DRUG-100227 |
| Methotrexate | GENERIC_DRUG-100152 |
| Vincristine | GENERIC_DRUG-102853 |
| Cisplatin | GENERIC_DRUG-104152 |

© 2020 Branigan GL et al. *JAMA Network Open.*

**eTable 4.** List of Diagnose Codes Used

| **Diagnosis** | **ICD-9** | **ICD-10** |
|---|---|---|
| Breast Cancer | ICD-9-D-1740, ICD-9-D-1741, ICD-9-D-1742, ICD-9-D-1743, ICD-9-D-1744, ICD-9-D-1745, ICD-9-D-1746, ICD-9-D-1748, ICD-9-D-1749, ICD-9-D-1750, ICD-9-D-1759, ICD-9-D-19881, ICD-9-D-217, ICD-9-D-2330, ICD-9-D-2383, ICD-9-D-2393, | ICD-10-D-C4352, ICD-10-D-C44501, ICD-10-D-C44511, ICD-10-D-C44521, ICD-10-D-C44591, ICD-10-D-C4A52, ICD-10-D-C50011, ICD-10-D-C50012, ICD-10-D-C50019, ICD-10-D-C50021, ICD-10-D-C50022, ICD-10-D-C50029, ICD-10-D-C50111, ICD-10-D-C50112, ICD-10-D-C50119, ICD-10-D-C50121, ICD-10-D-C50122, ICD-10-D-C50129, ICD-10-D-C50211, ICD-10-D-C50212, ICD-10-D-C50219, ICD-10-D-C50221, ICD-10-D-C50222, ICD-10-D-C50311, ICD-10-D-C50312, ICD-10-D-C50319, ICD-10-D-C50321, ICD-10-D-C50322, ICD-10-D-C50329, ICD-10-D-C50411, ICD-10-D-C50412, ICD-10-D-C50419, ICD-10-D-C50421, ICD-10-D-C50422, ICD-10-D-C50429, ICD-10-D-C50511, ICD-10-D-C50512, ICD-10-D-C50519, ICD-10-D-C50521, ICD-10-D-C50522, ICD-10-D-C50611, ICD-10-D-C50612, ICD-10-D-C50619, ICD-10-D-C50621, ICD-10-D-C50622, ICD-10-D-C50811, ICD-10-D-C50812, ICD-10-D-C50819, ICD-10-D-C50821, ICD-10-D-C50822, ICD-10-D-C50829, ICD-10-D-C50911, ICD-10-D-C50912, ICD-10-D-C50919, ICD-10-D-C50921, ICD-10-D-C50922, ICD-10-D-C50929, ICD-10-D-C7981, ICD-10-D-D0352, ICD-10-D-D0500, ICD-10-D-D0501, ICD-10-D-D0502, ICD-10-D-D0510, ICD-10-D-D0511, ICD-10-D-D0512, ICD-10-D-D0580, ICD-10-D-D0581, ICD-10-D-D0582, ICD-10-D-D0590, ICD-10-D-D0591, ICD-10-D-D0592, ICD-10-D-D241, ICD-10-D-D242, ICD-10-D-D249, ICD-10-D-D4860, ICD-10-D-D4861, ICD-10-D-D4862, ICD-10-D-D493 |
| NDD | See AD, Dementia, MS, Parkinson, and ALS Codes | |
| AD | ICD-9-D-3310 | ICD-10-D-G300, ICD-10-D-G301, ICD-10-D-G308, ICD-10-D-G309 |
| Dementia | ICD-9-D-2900, ICD-9-D-29010, ICD-9-D-29011, ICD-9-D-29012, ICD-9-D-29013, ICD-9-D-29020, ICD-9-D-29021, ICD-9-D-2903, ICD-9-D-29040, ICD-9-D-29041, ICD-9-D-29042, ICD-9-D-29043, ICD-9-D-29410, ICD-9-D-29411, ICD-9-D-29420, ICD-9-D-29421, ICD-9-D-33119, ICD-9-D-33182 | ICD-10-D-F0150, ICD-10-D-F0151, ICD-10-D-F0280, ICD-10-D-F0281, ICD-10-D-F0390, ICD-10-D-F0391, ICD-10-D-G3109, ICD-10-D-G3183 |
| MS | ICD-9-D-340 | ICD-10-D-G35 |
| Parkinson's | ICD-9-D-332, ICD-9-D-3320 | ICD-10-D-G20, ICD-10-D-G214 |
| ALS | ICD-9-D-33520 | ICD-10-D-G1221 |
| T2D | ICD-9-D-25000, ICD-9-D-25001, ICD-9-D-25002, ICD-9-D-25003 | ICD-10-D-E108, ICD-10-D-E109, ICD-10-D-E1169, ICD-10-D-E118, ICD-10-D-E119 |
| HTN | ICD-9-D-4010, ICD-9-D-4011, ICD-9-D-4019 | ICD-10-D-I10, ICD-10-D-I110, ICD-10-D-I119 |
| COPD | ICD-9-D-49120, ICD-9-D-49121, ICD-9-D-49122, ICD-9-D-49320, ICD-9-D-49321, ICD-9-D-49322 | ICD-10-D-J440, ICD-10-D-J441, ICD-10-D-J449 |
| CKD | ICD-9-D-5851, ICD-9-D-5852, ICD-9-D-5853, ICD-9-D-5854, ICD-9-D-5855, ICD-9-D-5859 | ICD-10-D-N181, ICD-10-D-N182, ICD-10-D-N183, ICD-10-D-N184, ICD-10-D-N185, ICD-10-D-N189 |
| CVD | ICD-9-D-4149, ICD-9-D-4280, ICD-9-D-4281, ICD-9-D-42820, ICD-9-D-42821, ICD-9-D-42822, ICD-9-D-42823, ICD-9-D-42830, ICD-9-D-42831, ICD-9-D-42832, ICD-9-42833, ICD-9-D-42840, ICD-9-D-42841, ICD-9-D-42842, ICD-9-D-42843, ICD-9-D-4289, ICD-9-D-4292 | ICD-10-D-I25700,ICD-10-D-I25708, ICD-10-D-I25709, ICD-10-D-I25710, ICD-10-D-I25719, ICD-10-D-I25720, ICD-10-D-I25729, ICD-10-D-I25739, ICD-10-D-I25798, ICD-10-D-I25799, ICD-10-D-I25810, ICD-10-D-I25811, ICD-10-D-I2582, ICD-10-D-I2583, ICD-10-D-I2584, ICD-10-D-I2589, ICD-10-D-I259, ICD-10-D-I501, ICD-10-D-I5043, ICD-10-D-I509 |
| Stroke | ICD-9-D-430, ICD-9-D-431, ICD-9-D-432, ICD-9-D-4320, ICD-9-D-4321, ICD-9-D-4329, ICD-9-D-43300, ICD-9-D-4331, ICD-9-D-43310, ICD-9-D-43311, ICD-9-D-43320, ICD-9-D-43321, ICD-9-D-43330, ICD-9-D-43331, ICD-9-D-43381, ICD-9-D-43390, ICD-9-D-43391, ICD-9-D-43400, ICD-9-D-43401, ICD-9-D-43410, ICD-9-D-43411, ICD-9-D-43490, ICD-9-D-43491 | ICD-10-D-I6300, ICD-10-D-I63011, ICD-10-D-I63012, ICD-10-D-I63031, ICD-10-D-I63032, ICD-10-D-I6310, ICD-10-D-I63139, ICD-10-D-I63232, ICD-10-D-I63233, ICD-10-D-I63239, ICD-10-D-I63312, ICD-10-D-I6339, ICD-10-D-I6340, ICD-10-D-I63411, ICD-10-D-I63432, ICD-10-D-I6350, ICD-10-D-I63512, ICD-10-D-I63519, ICD-10-D-I63529, ICD-10-D-I6359, ICD-10-D-I638, ICD-10-D-I639 |

© 2020 Branigan GL et al. *JAMA Network Open.*

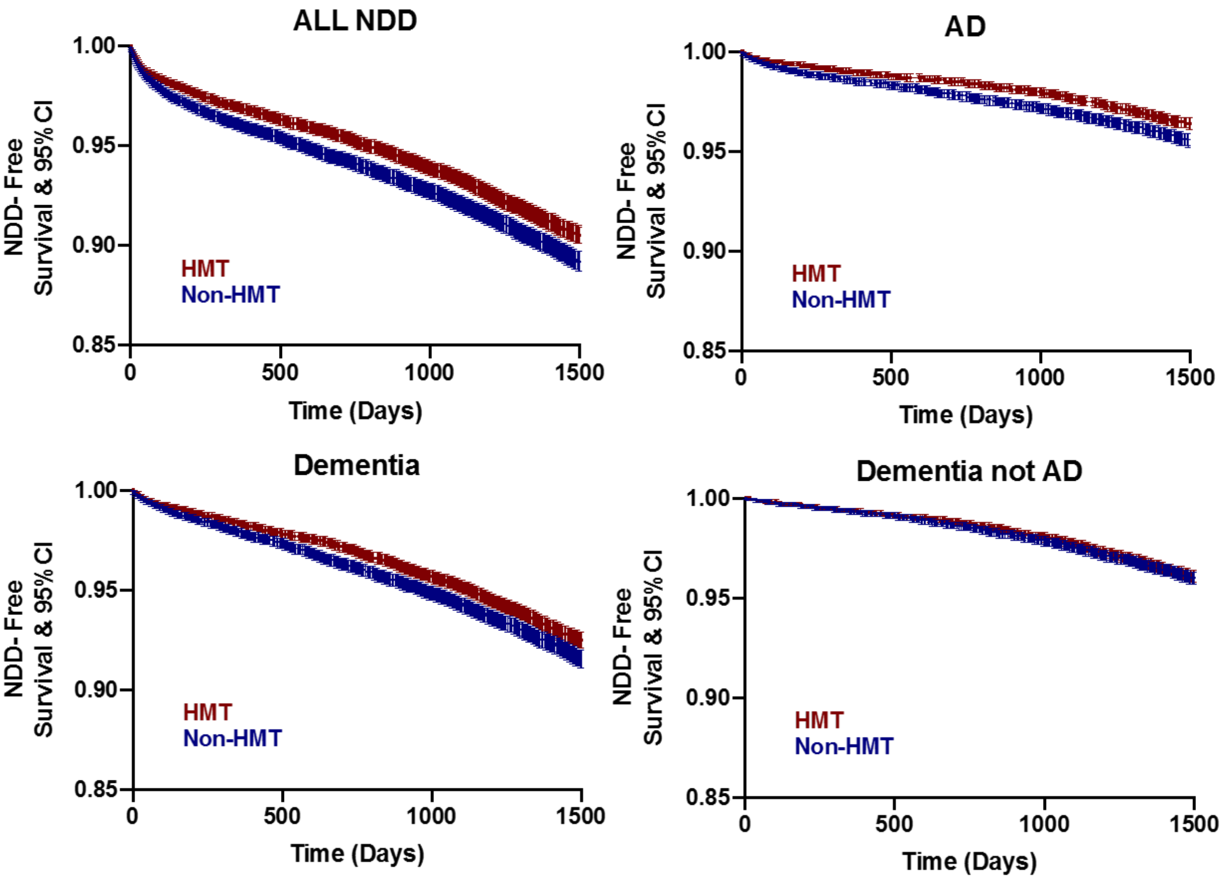


**eFigure 1. Reduced risk of all NDD, AD and Dementia in propensity score matched patients with HMT exposure across all age groups.**

Abbreviations: HMT, hormone modulating therapy; All NDD, All Neurodegenerative Disease Composite Group; AD, Alzheimer's Disease; Dem, dementia; Dem no AD, non-Alzheimer's type dementia

© 2020 Branigan GL et al. *JAMA Network Open.*

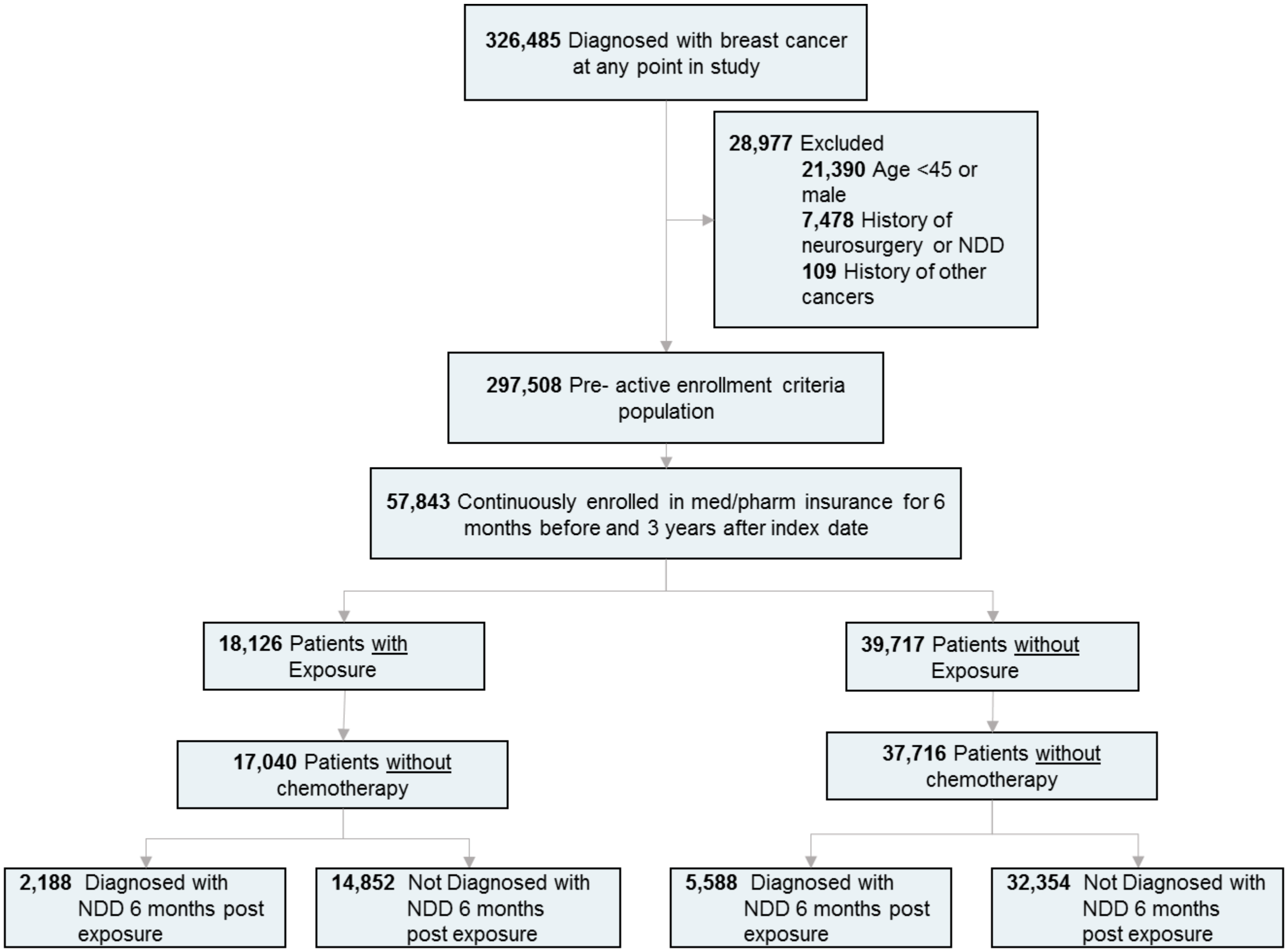


**eFigure 2. Patient breakdown without chemotherapy**

Abbreviations: DOI, drug of interest; NDD, neurodegenerative disease

© 2020 Branigan GL et al. *JAMA Network Open.*

| | *Unadjusted without Chemotherapy* | | | |
|---|---|---|---|---|
| | **Patients with HMT exposure** | | | |
| | **ALL NDD** | **AD** | **Dem** | **Dem not AD** |
| **# Patients** | 2,188 | 878 | 1,823 | 1,016 |
| **%** | *12.84* | *5.15* | *10.70* | *5.96* |
| | **Patients without HMT exposure** | | | |
| **# Patients** | 5,588 | 2,362 | 4,734 | 2,529 |
| **%** | *14.82* | *6.62* | *12.55* | *6.71* |
| **Relative Risk** | 0.87 | 0.82 | 0.85 | 0.89 |
| **95%CI** | 0.83 to 0.91 | 0.76 to 0.89 | 0.81 to 0.90 | 0.83 to 0.95 |
| **NNT** | 50.62 | 90.09 | 53.96 | 134.6 |
| ***p-value*** | <0.001 | <0.001 | <0.001 | 0.001 |

**eTable 5. Relative risk of patients without chemotherapy taking HMT to develop NDDs**

Abbreviation: HMT, hormone modulating therapy; AD, Alzheimer's Disease; Dem, dementia; Dem no AD, non-Alzheimer's type dementia

© 2020 Branigan GL et al. *JAMA Network Open.*

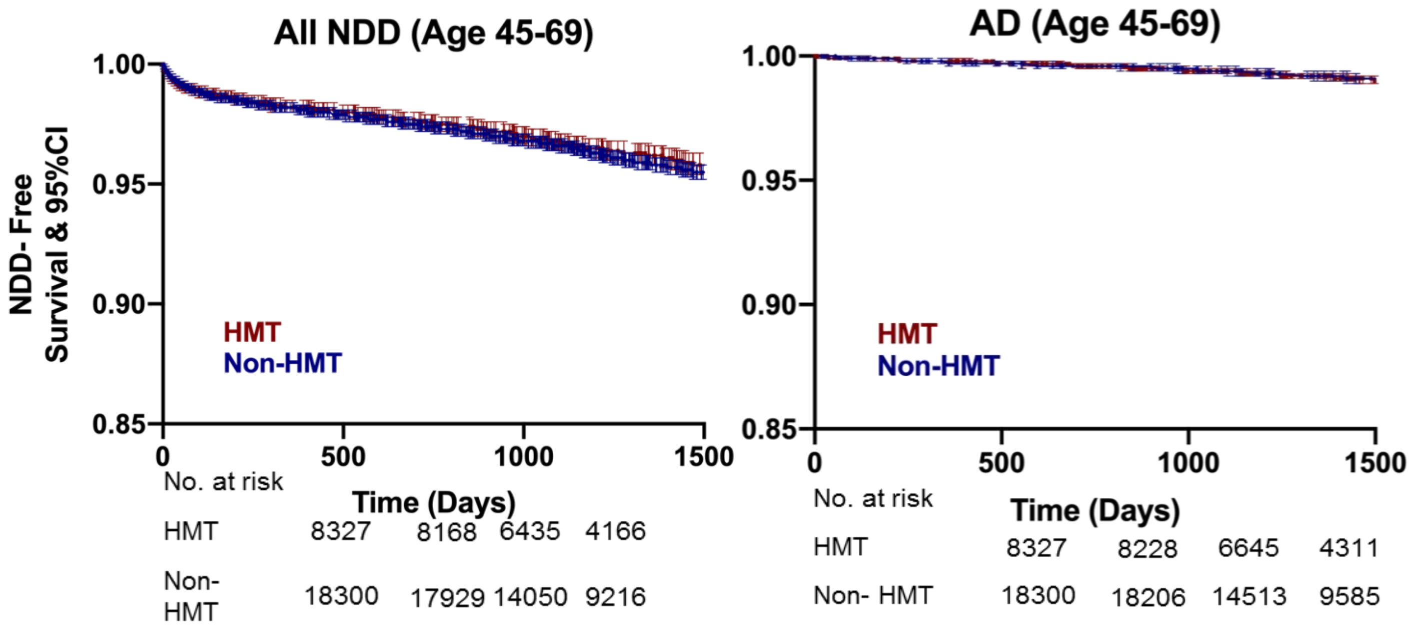


**eFigure 3. Age-dependent reduction in risk for All NDD and Alzheimer's Disease is not associated with HMT exposure for patients less than 70 years of age.**

Abbreviations: Non-HMT, Patients not exposed to hormone modulating therapy (HMT); HMT, Patients exposed to HMT

© 2020 Branigan GL et al. *JAMA Network Open.*

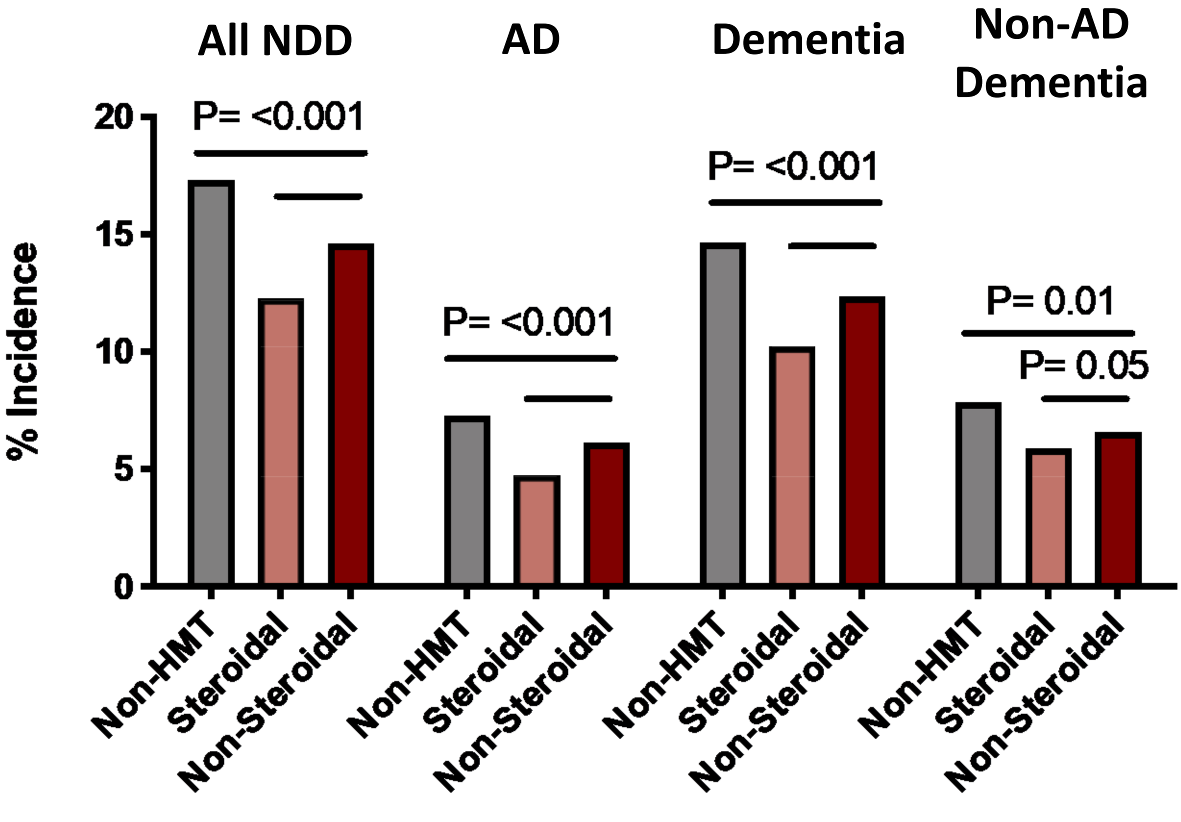


**eFigure 4. Steroidal aromatase inhibitors are associated with the protective effects seen within the aromatase inhibitor group.**

Abbreviations: Non-HMT, Patients not exposed to hormone modulating therapy (HMT); Steroidal, Steroidal Aromatase inhibitor (exemestane); Non-Steroidal, Non-steroidal aromatase inhibitors (anastrozole and letrozole)

© 2020 Branigan GL et al. *JAMA Network Open.*
